# Резолюция национального междисциплинарного совета экспертов «Высокодозный витамин D (Девилам) в практике акушера-гинеколога»

**DOI:** 10.14341/probl13465

**Published:** 2024-05-09

**Authors:** Е. Н. Андреева, Н. В. Артымук, А. Ф. Веснина, И. Е. Зазерская, Л. Ю. Карахалис, Н. Ю. Каткова, Е. А. Пигарова, И. В. Сахаутдинова, Н. В. Спиридонова, Н. И. Тапильская, М. Б. Хамошина, Е. В. Шереметьева, С. В. Юренева, М. И. Ярмолинская

**Affiliations:** Национальный медицинский исследовательский центр эндокринологии; Российский университет медицины; Кемеровский государственный медицинский университет; Национальный медицинский исследовательский центр эндокринологии; Национальный медицинский исследовательский центр им. В. А. Алмазова; Кубанский государственный медицинский университет; Приволжский исследовательский медицинский университет; Национальный медицинский исследовательский центр эндокринологии; Башкирский государственный медицинский университет; Самарский государственный медицинский университет; Научно-исследовательский институт акушерства, гинекологии и репродуктологии имени Д.О. Отта; Российский университет дружбы народов; Национальный медицинский исследовательский центр эндокринологии; Национальный медицинский исследовательский центр акушерства, гинекологии и перинатологии имени В.И. Кулакова; Научно-исследовательский институт акушерства, гинекологии и репродуктологии имени Д.О. Отта

**Keywords:** 25-ОН-D, витамин D, гинекология, акушерство

## Abstract

28 марта 2024 года в Москве состоялся Совет Экспертов «Высокодозовый витамин D (Девилам) в практике акушерагинеколога, гинеколога и эндокринолога» с участием ведущих экспертов гинекологов, эндокринологов и акушеровгинекологов, в рамках которого были обсуждены новые возможности применения высокодозного витамина D у пациенток различных возрастов, нуждающихся в коррекции имеющихся дефицита или недостаточности витамина D.

## Abstract (en)

On March 28, 2024, the Council of Experts “High-dose vitamin D (Devilam) in the practice of obstetrician-gynecologist, gynecologist and endocrinologist” was held in Moscow with the participation of leading experts gynecologists, endocrinologists and obstetricians-gynecologists, during which new possibilities for the use of high-dose vitamin D in patients of various ages who need correction of existing vitamin D deficiency or insufficiency.

Дефицит витамина D — это состояние, характеризующееся снижением концентрации 25(ОН)D в крови ниже оптимального уровня, которое может приводить к снижению всасывания кальция в кишечнике, развитию вторичного гиперпаратиреоза и повышению риска переломов, особенно у пожилых лиц [1][2].

В последние десятилетия значительный интерес представляет подробное изучение физиологической роли витамина D в организме, его значения в функционировании тканей и органов, а также вклада, который вносит изменение его метаболизма в развитие хронических заболеваний, течение и исход беременности [3].

В настоящее время дефицит витамина D принял характер пандемии XXI века, затрагивающей все континенты и типы пациентов, независимо от места проживания, пола, возраста, социального статуса и расы [4–9].

Публикации последних лет свидетельствуют, что эта проблема актуальна не только для специалистов-эндокринологов, терапевтов и врачей общей практики, но и в целом в современном акушерстве и гинекологии. Многочисленные исследования, включая метаанализы последних лет, подтверждают влияние недостаточного уровня витамина D на репродуктивное здоровье женщины, включая здоровье ее потомства [10–14 ]. А также существуют исследования, которые показали положительное влияние витамина D на здоровье мужчины и его роль на фертильность мужской половины человечества [15–18].

Кроме того, и женщины, находящиеся в периоде пери- и постменопаузы, подвержены негативному риску влияния на общее здоровье, сердечно-сосудистую, общую смертность и смертность, связанную с онкологическими заболеваниями, связанными с дефицитом или недостаточностью витамина D [19–23].

Общепризнанным является значение витамина D не только в качестве вещества, участвующего в кальциево-фосфорном обмене, и, как следствие, в развитии остеопороза и связанных с ним переломов и падений, особенно у женщин старших возрастных категорий, когда продукция и защитные функции женских половых гормонов значимо снижаются [24–26].

Таким образом, риску развития патологических состояний, связанных с дефицитом/недостаточностью витамина D, подвержены женщины различных возрастов.

## РОЛЬ ВИТАМИНА D В ПРОФИЛАКТИКЕ ГЕСТАЦИОННЫХ ОСЛОЖНЕНИЙ НА ЭТАПЕ ПОДГОТОВКИ К БЕРЕМЕННОСТИ

Важное значение недостаточность/дефицит витамина D приобретает в прегравидарный период и в период беременности [27]. Современные представления о плейотропных эффектах витамина D позволяют предположить, что из-за недостатка этого вещества может снижаться вероятность наступления беременности либо, в случае ее наступления, увеличивается риск развития различных патологий [28]. Анализ многочисленных исследований свидетельствует о неблагоприятном влиянии дефицита витамина D на течение и исход беременности.

С его дефицитом ассоциированы риски развития гестационного сахарного диабета, преэклампсии, плацентарной недостаточности, низкой массы тела при рождении, преждевременных родов, бактериальных инфекций [29–32]. Следовательно, необходимо определить адекватную дозировку колекальциферола, которая позволит компенсировать как непосредственно дефицит витамина D, так и возможное отрицательное влияние на течение беременности.

Совет экспертов рекомендует внести в Клинические рекомендации по прегравидарной подготовке к беременности определение уровня сывороточного 25(ОН)D как минимум за 3 месяца до планируемой беременности у женщин из группы риска по тяжелому дефициту витамина D, чтобы в случае выявленного дефицита/недостаточности витамина D иметь возможность компенсировать данное состояние, включая использование возможностей скорости насыщения организма пациентки высокодозным витамином D «Девилам» [33].

## Дефицит витамина D и исходы вспомогательных репродуктивных технологий (ВРТ)

Повышение эффективности методов вспомогательных репродуктивных технологий (ВРТ) — актуальная задача современной репродуктивной медицины. Исследования последних лет показывают четкую взаимосвязь между уровнем витамина D и исходами беременности в программах ВРТ. Витамин D играет важную роль в поддержании репродуктивного здоровья женщин. Рецепторы к витамину D обнаружены во многих структурах репродуктивной системы: в яичниках (особенно в гранулезных клетках), эндометрии, эпителии маточных труб, плаценте, децидуальных клетках, гипофизе и гипоталамусе [34]. Биологические эффекты витамина D и прогестерона синергичны — они способствуют поддержанию беременности за счет индукции противовоспалительных путей (Th2- и Treg-клеток), стимуляции высвобождения кортикотропин-рилизинг-фактора (CRF), активина А, фактора роста сосудистого эндотелия (VEGF), остеопонтина, кальбиндина, ингибировании провоспалительных путей (NK, Th1- и Th17-клетки), регуляции эндометриальной экспрессии гена HOXA10, участвующего в процессах имплантации эмбриона [35].

Главные доказательства участия витамина D в репродуктивных процессах были получены в исследованиях с использованием ВРТ, показавших снижение уровней наступления клинической беременности в результате негативного влияния дефицита D на рецептивность эндометрия [36–39]. По данным метаанализов 2018 г. было отмечено:

-при дефиците витамина D наблюдается достоверное снижение уровня живорождения после циклов ВРТ по сравнению с женщинами с нормальным уровнем витамина D;

-вероятность наступления клинической беременности оказалась статистически значимо выше в группе женщин с адекватным уровнем витамина D, как и вероятность рождения живых детей, по сравнению с женщинами с недостаточностью или дефицитом витамина D [40][41].

В руководящих документах РФ подчеркнута необходимость скрининга уровня витамина D у пациентов группы риска [42][43]. С учетом представленных данных выше женщины с бесплодием, с несколькими неудачными попытками ЭКО, а также планирующих протоколы ВРТ, тоже могут быть отнесены к группе риска. В рамках подготовки к процедурам ВРТ следует помнить о возможном дефиците витамина D или его недостаточном уровне и необходимости коррекции выявленных нарушений, особенно у женщин, входящих в группы повышенного риска (например, пациентки с ожирением, бариатрическими операциями в анамнезе и т.д.).

Совет экспертов особо отметил важность назначения исследования на недостаточность/дефицит витамина D в рамках подготовки к процедурам ЭКО/ВРТ не только для женщины, но и обязательное исследование, а при обнаружении — и лечение недостаточности/дефицита витамина D для мужчины в бесплодной паре. Если семья планирует ВРТ, на этапе подготовки к процедуре необходимо назначение определения уровня 25(OH)D, кальция и альбумина в биохимическом анализе крови для обоих партнеров.

## Дефицит/недостаточность витамина D у женщин с синдромом поликистозных яичников

Синдром поликистозных яичников (СПЯ) — полигенное эндокринное расстройство, обусловленное как генетическими, так и эпигенетическими факторами, при этом ожирение и инсулинорезистентность (ИР) часто встречаются при этой патологии и усугубляют течение самого заболевания. Во многих исследованиях выявлена обратная связь между обеспеченностью витамином D и метаболическими нарушениями при СПЯ. Недостаточность витамина D может являться фактором риска нарушения толерантности к глюкозе, ИР и сахарного диабета 2 типа (СД2) [44].

Ожирение коррелирует с более низким уровнем 25(OH)D в основном в результате разрушения липофильного витамина в жировой ткани [45]. Механизм действия витамина D при ожирении связан с влиянием на гены регуляции углеводного и жирового метаболизма [46]. Получены данные о высокой распространенности дефицита витамина D среди женщин с СПЯ и его обратной корреляции с маркерами чувствительности к инсулину [47][48]. В систематический обзор и метаанализ эффекта применения Ca/витамина D у женщин с СПЯ [49] было включено 6 рандомизированных контролированных исследований (n=280), авторы отметили, что оптимальный уровень витамина D способствует:

Таким образом, витамин D может быть использован в качестве дополнительного препарата, способствующего улучшению чувствительности тканей к инсулину и улучшающего метаболизм у женщин с СПЯ.

Адекватный статус витамина D имеет большое значение для успешной овуляции и повышения частоты наступления беременности у страдающих бесплодием женщин с СПЯ. В исследованиях было показано:

## Дефицит витамина D и эндометриоз

Эндометриоз — это хроническое, рецидивирующее, прогрессирующее, гормонозависимое заболевание, характеризующееся доброкачественным разрастанием за пределами полости матки ткани, сходной по морфологической структуре и функциям с эндометрием. Эндометриоз, несомненно, является мультифакторным заболеванием, загадочным и многоликим в своих клинических проявлениях, которое диагностировано более чем у 256 миллионов женщин в мире, ассоциировано с бесплодием у 35–50% пациенток, с хроническим болевым синдромом — у 70–80% женщин [52, 53]. Эндометриоз-ассоциированный хронический болевой синдром является серьезной проблемой и может отрицательно влиять на психоэмоциональное состояние и качество жизни пациенток [54]. Не существует единого подхода и универсального метода лечения, который гарантировал бы полное излечение и отсутствие рецидивов заболевания [55].

Данные относительно ассоциации уровня 25(OH)D и эндометриоза довольно противоречивы. Результатов многочисленных исследований на данном этапе недостаточно для доказательства роли витамина D в патогенезе генитального эндометриоза, а для применения колекальциферола в качестве нового подхода в терапии заболевания необходимо проведение дополнительных исследований [56]. В работе Miyashita M. и соавт. отмечены более низкие уровни 25(OH)D в сыворотке крови у больных с распространенным эндометриозом по сравнению с заболеванием средней степени тяжести у здоровых женщин [57], в то время как Somigliana E. и соавт., напротив, обнаружили более высокие концентрации сывороточного 25(OH)D в группе женщин с эндометриозом, при этом средний уровень 25(ОН)D у больных эндометриозом составил 24,9±14,8 нг/мл, что, согласно референсным значениям, относится к недостаточности его содержания [58]. Таким образом, ряд исследователей утверждает, что существенной связи между уровнем 25(ОН)D и эндометриозом не выявлено [59–62]; между тем другие работы демонстрируют значительно более низкие уровни 25(ОН)D в сыворотке крови в группах пациенток с эндометриозом [63-67].

Baek J.C. и соавт. [65] отметили достоверную корреляцию между уровнем 25(ОН)D и тяжестью заболевания, а Anastasi E. и соавт. [66], напротив, не выявили зависимости между концентрацией 25(ОН)D в сыворотке крови и степенью распространенности заболевания, бесплодием или индексом массы тела, но обнаружили значимую корреляцию между низким уровнем 25(OH)D и наличием любого типа эндометриоз-ассоциированного болевого синдрома с выраженностью ≥5 баллов по ВАШ. Также исследователи показали, что в группе женщин с эндометриозом средний уровень сывороточного 25(ОН)D составлял 21,3±8,9 нг/мл: дефицит 25(ОН)D наблюдался у 48%, недостаточность — у 32%, и только у 20% женщин отмечено содержание в пределах референсных значений. Дефицит/недостаточность 25(ОН)D значительно чаще диагностировались у больных эндометриозом по сравнению со здоровыми женщинами (80 против 33,3%; р<0,001) [63]. Ciavatini A. и соавт. отметили достоверную линейную корреляцию между уровнем витамина D в сыворотке крови и диаметром эндометриоидных кист. У 85,7% обследованных женщин с односторонней эндометриомой был диагностирован сниженный уровень витамина D в сыворотке крови (уровень 25(ОН)D ниже 30 нг/мл), при этом в данной группе больных средний диаметр кисты яичника был практически в 2 раза больше, чем у пациенток c эндометриоидными кистами и уровнем витамина D в пределах референсных значений [68]. Российские исследователи также утверждают, что для женщин с эндометриозом яичников характерно снижение концентрации 25(ОН)D до значений, соответствующих критериям недостаточности (23,98±6,82 нг/мл), а при наличии выраженной тазовой боли — до 19,26±6,01 нг/мл, что соответствует критериям дефицита. Также была отмечена достоверная умеренная обратная корреляция между интенсивностью тазовой боли, обусловленной эндометриозом яичников, и уровнем витамина D в крови [69].

Известно, что рецептор витамина D (VDR) кодируется геном рецептора витамина D (VDR). Наиболее значимыми и исследованными полиморфными вариантами гена VDR, ассоциированными с развитием ряда заболеваний, являются: rs1544410 (BsmI), rs2228570 (FokI), rs731236 (TaqI), rs7975232 (ApaI). Известно, что частота аллеля G полиморфного варианта rs1544410 (BsmI) гена VDR достоверно выше у пациенток с наружным генитальным эндометриозом (НГЭ) по сравнению с популяционной выборкой. Установлено наличие достоверных различий для генотипа G/G полиморфного варианта rs1544410 (BsmI) гена VDR у больных НГЭ относительно группы контроля (p<0,05), согласно коэффициенту соотношения шансов риск развития НГЭ в 1,9 раза выше при данном генотипе (OR=1,93 CI=1,082-3,450). Сочетание генотипов A/A+G/A, напротив, достоверно чаще встречается в популяции (p=0,025) по сравнению с пациентками с генитальным эндометриозом [70].

VDR экспрессируются как в эндометриоидных гетеротопиях, так и в эндометрии больных НГЭ, а также в эндометрии здоровых женщин. Zelenko Z. и соавт. обнаружили, что экспрессия VDR ниже в среднюю секреторную по сравнению с ранней секреторной фазой менструального цикла как у женщин с НГЭ, так и в контрольной группе [71]. Bergadà L. и соавт. представили данные иммуногистохимического анализа, которые показали снижение уровня экспрессии VDR у больных карциномой эндометрия (n-157) по сравнению с нормальным эндометрием (n-60) [72]. Известно, что эндометриоидные гетеротопии характеризуются сниженным уровнем экспрессии VDR по сравнению с эндометрием больных НГЭ и эндометрием контрольной группы в секреторную фазу менструального цикла. При этом в эндометриоидных гетеротопиях уровень экспрессии VDR сопоставим со значениями в эндометрии контрольной группы в пролиферативную фазу менструального цикла. Экспрессия VDR у здоровых женщин в эндометрии в 1,5 раза выше в секреторную фазу менструального цикла по сравнению с пролиферативной фазой, то есть имеет циклические изменения. У больных НГЭ, напротив, не выявлено циклических изменений экспрессии VDR в эндометрии, что, возможно, имеет важное значение в патогенезе заболевания, может свидетельствовать об изменении рецептивности эндометрия, нарушениях имплантации и быть одной из причин эндометриоз-ассоциированного бесплодия. Однако необходимо проведение дальнейших исследований в данном направлении [73].

Результаты работ на экспериментальных моделях — мышах и крысах — хирургически-индуцированного эндометриоза демонстрируют значительное снижение площади эндометриоидных гетеротопий и, в некоторых случаях, полную резорбцию поражений на фоне проводимой терапии колекальциферолом и его селективным агонистом элокальцитолом [74–78]. Также в этих работах продемонстрирована способность колекальциферола и элокальцитола оказывать неклассические эффекты, среди которых: противовоспалительный эффект за счет снижения количества макрофагов и уровня провоспалительного цитокина интерлейкина-1 (IL-1) в перитонеальной жидкости [74]; антиапоптотический — развитие апоптоза в стромальном компоненте имплантатов [75] и антиангиогенный за счет снижения уровней VEGF (васкулоэндотелиального фактора роста), матриксной металлопротеиназы-9 (MMP-9) и возможности повышать содержание TIMP-2 (тканевой ингибитор металлопротеиназы-2) [76].

Данные относительно влияния колекальциферола на болевой синдром носят противоречивый характер. При проведении двойного слепого клинического исследования Almassinokiani F. и соавт. не обнаружили существенных различий в уменьшении выраженности хронической тазовой боли и дисменореи после лечения колекальциферолом или плацебо [79]. Lasco A. и соавт. в своем исследовании, напротив, установили, что колекальциферол при однократном приеме в дозе 300 000 МЕ за 5 дней до предполагаемой менструации по сравнению с плацебо у женщин с первичной дисменореей, ассоциированной с эндометриозом, статистически значимо уменьшает выраженность болевого синдрома [80]. Снижение интенсивности болевого синдрома, вероятно, связано со способностью кальцитриола (путем подавления циклооксигиназы-2) влиять на синтез простагландинов в эндометрии и инактивировать их вследствие усиления регуляции 15-гидроксипростагландин-дегидрогеназы [81]. Также известно, что сочетанное применение аГнРГ 3,75 мг или диеногеста 2 мг с колекальциферолом способствует более выраженному уменьшению болевого синдрома и стабилизации психоэмоционального фона по сравнению со стандартной гормономодулирующей терапией [67][82][83].

## Антипролиферативные свойства витамина D

Антипролиферативные свойства витамина D оказывают влияние как на состояние эпидермиса и волосяных фолликулов, так и на их способность к восстановлению после повреждения УФ-излучением. Доказанным считается влияние на подавление пролиферации эпителиальных клеток при раке молочной железы (РМЖ), толстой кишки, простаты [84]. У женщин в постменопаузе с раком молочной железы наблюдалась связь между недостаточностью или дефицитом витамина D и опухолями с худшими прогностическими признаками РМЖ [85].

У коморбидной пациентки на приеме у акушера-гинеколога выявление дефицита или недостаточности витамина D представляется крайне важным для его своевременного и эффективного восполнения с целью повышения качества жизни, качественной прегравидарной подготовки и ведения беременности, комбинированной терапии СПЯ, онкопротекции относительно РМЖ, снижения риска развития депрессии, сердечно-сосудистых заболеваний (ССЗ), а также снижения риска развития остеопении и саркопении у пожилых пациенток. Два последних состояния, с учетом их достаточно медленного и малосимптомного развития, способны привести к развитию таких осложнений, как частые падения и низкоэнергетические переломы крупных костей, что резко ухудшает прогноз для пожилого пациента [84][87–94].

## Дефицит витамина D и менопауза

Дефицит витамина D оказывает негативное влияние на течение переходного периода женщины и в период менопаузы. Дефицит витамина D составляет 31–70% у женщин в постменопаузе. Женщины в постменопаузе подвержены повышенному риску ССЗ. Уровни 25(OH)D в сыворотке связаны с такими важными факторами риска ССЗ, как СД, высокий уровень триглицеридов, гипертония, ожирение и высокий риск смертности. Таким образом, существует связь между менопаузой, низким содержанием витамина D в сыворотке и сердечно-сосудистыми заболеваниями [95–98].

В исследовании NHANES (2023 г.) отмечено, что дефицит и недостаточность витамина D могут быть связаны с более ранним возрастом наступления менопаузы [95].

К плейотропным эффектам витамина D в период менопаузы можно отнести: профилактику саркопении, снижение рисков ССЗ, протекцию в рамках генитоуринарного менопаузального синдрома (ГУМС), купирование вазомоторных симптомов, модулирование иммунной функции и влияние на выработку адипокинов, а также антипролиферативное действие на опухолевые клетки [99][100].

Распространенность метаболического синдрома резко увеличивается с наступлением менопаузы и может достигать 30–70% по сравнению с 14–45% у женщин репродуктивного возраста [101][102]. У женщин в постменопаузе с дефицитом витамина D прием 1000 МЕ/сут в течение 9 месяцев способствовал снижению риска метаболического синдрома [103]. Витамин D способствует компенсации нарушений углеводного обмена у женщин в постменопаузе [104].

Женщины с низким уровнем витамина D имеют больше риска развития пролапса тазовых органов. Более того, уровень витамина D был обратно пропорционален тяжести пролапса тазовых органов [105][106]. Повышение уровня витамина D было связано с уменьшением риска расстройств тазового дна, улучшением среднего модифицированного индекса здоровья влагалища у женщин в возрастной группе 65–78 лет [107].

Для успешного формирования настороженности у акушеров-гинекологов необходимо привлечь их внимание к возможному риску возникновения дефицита/недостаточности витамина D у пациенток различных возрастных категорий с наиболее часто встречающимися у специалиста на приеме заболеваниями. Понятный акушеру-гинекологу портрет пациентки позволит в условиях дефицита времени максимально эффективно принять решение, запланировать обследование и сделать назначения, направленные на устранение выявленных нарушений.

Таким образом, с учетом необходимости быстрого и простого выбора пациенток, нуждающихся в скрининге для определения сывороточного витамина D, оптимальным представляется формирование перечня основных портретов пациенток, для которых установлен высокий риск развития дефицита/недостаточности витамина D:

Витамин D может быть полезен при вагинальном дискомфорте у женщин в период менопаузы [107][115], однако некоторые экспериментальные результаты противоречивы [118][119]. В исследовании 2017 г. было показано, что витамин D обладает фунгицидной активностью в отношении Candida Albicans. Исследование проводилось с помощью антимикробного скрининга с использованием модифицированного агара методом диффузии. Механизм противогрибкового действия может быть связан с повышенной жирорастворимостью витамина D, способного изменять целостность клеточной мембраны [120].

Результаты исследования показали, что чем выше уровень витамина D, тем ниже риск возникновения дисфункции тазового дна у женщин [121][122], дислипидемии, метаболического синдрома, сахарного диабета 2 типа.

Совет экспертов особо отметил необходимость скрининга на дефицит витамина D и внесения в стандарты ОМС и Клинические рекомендации положения о праве врача акушера-гинеколога назначать исследование уровня витамина D в сыворотке крови, а также вместе с определением уровня витамина D определять уровень общего кальция и альбумина в биохимическом анализе крови с расчетом уровня кальция, скорректированного на альбумин, у пациенток, предрасположенных к развитию недостаточности/дефицита витамина D:

Согласно проекту «Клинические рекомендации по диагностике, лечению и профилактике дефицита витамина D у взрослых» Российской ассоциации эндокринологов, критериями недостаточности/дефицита витамина D являются [1]:

## Клиническая фармакология, эффективность и безопасность приема высокодозового витамина D

Девилам — новый высокодозный препарат витамина D c содержанием колекальциферола 5000 МЕ или 50 000 МЕ в одной таблетке. Препарат Девилам имеет преимущества формы выпуска (матричной таблетки), что позволяет обеспечивать сохранность от воздействия факторов окружающей среды и более равномерное всасывание колекальциферола, точность дозирования и высокую биодоступность лекарственного препарата [33][123].

Форма выпуска препарата Девилам в виде матричной таблетки представляет в своей основе матричный каркас — многоуровневую ячеистую структуру из натурального желатина. Из порошка колекальциферола формируются сыпучие микрочастицы, покрытые липидными комплексами и путем напыления под высоким давлением колекальциферол, покрытый липидными комплексами, в виде «бусин» помещается в ячейки матрикса. Множество матричных слоев соединяются между собой и формируют таблетку. Таблетка покрывается снаружи пленочной оболочкой, резистентной к воздействию кислой среды желудочного сока (рис. 1).

**Figure:**
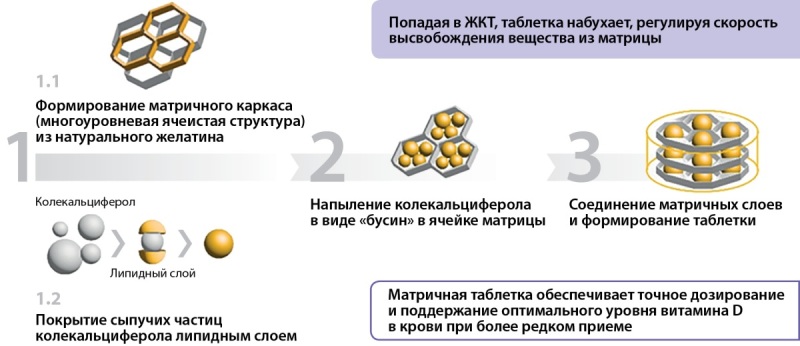
Рисунок 1. Этапы формирования матричной таблетки Девилам. Figure 1. Stages of formation of the Devilam matrix tablet.

Таким образом, выход колекальциферола из матричной таблетки происходит в тонком кишечнике путем медленной диффузии из матричного каркаса покрытых липидной оболочкой молекул колекальциферола, что как раз и позволяет обеспечить равномерность всасывания и точность дозирования препарата (рис. 2).

**Figure:**
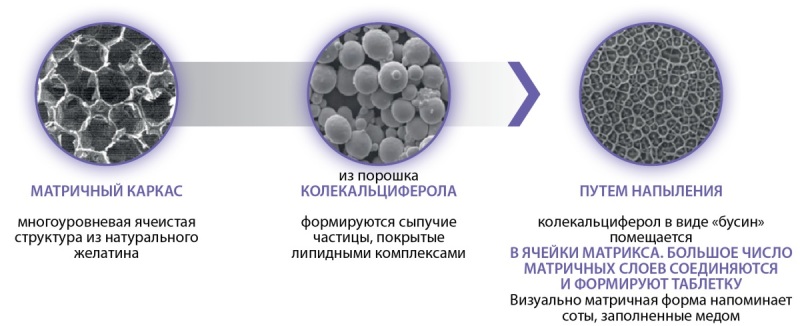
Рисунок 2. Структура матричной таблетки Девилам под увеличением. Figure 2. Structure of Devilam matrix tablet under magnification

Исследования показали, что прием высоких доз колекальциферола 50 000 МЕ более эффективно и быстро повышает уровни 25(ОН)D в сыворотке крови, чем при низкодозных курсах [124][125].

С точки зрения безопасности было показано, что высокодозная схема приема витамина D безопасна и эффективна у коморбидных пациентов с метаболическим синдромом [126].

Эксперты постановили, что препараты колекальциферола (в частности, препарат Девилам) в форме матричной таблетки с дозировкой 50 000 МЕ и 5 000 МЕ могут быть востребованы в РФ в рамках профилактики и устранения недостаточности/дефицита витамина D (табл. 1).

**Table:** Таблица 1. Примеры режимов дозирования препарата Девилам* для профилактики и лечения дефицита витамина D Table 1. Examples of Devilam* dosage regimens for the prevention and treatment of vitamin D deficiency * — лекарственный препарат Девилам противопоказан при беременности и грудном вскармливании, поскольку не проводились рандомизированные клинические исследования у данных когорт пациенток.

Профилактика дефицита/недостаточности витамина D: Колекальциферол 800–4000 МЕ в сутки ИЛИ 5000–30 000 МЕ в неделю ИЛИ 25 000–50 000 в месяц	Девилам 5000 через день ИЛИ Девилам 5000 от 1 до 6 таблеток в неделю	Девилам 50 000 1 таблетка 1 раз в месяц ИЛИ 1 таблетка 1 раз в 2 недели в зависимости от массы тела пациента
Профилактика дефицита/недостаточности витамина D у лиц с морбидным ожирением или тяжелым нарушением функции кишечником 5000–10 000 МЕ в сутки	Девилам 5000 МЕ ежедневно	Девилам 50 000 МЕ 1 таблетка 1 раз в 2 недели
Лечение установленного дефицита/недостаточности витамина D (<30 нг/мл)	Девилам 10 таб. По 5000 МЕ еженедельно	Девилам 50 000 МЕ 1 таб. 1 раз в неделю в течение 4 недель при уровне витамина D (20–30 нг/мл), в течение 8 недель при уровне витамина D <20 нг/мл, далее перейти на поддерживающие дозы колекальциферола

В таблице 1 представлены варианты приема таблеток Девилам на рекомендуемые профилактические и лечебные дозы колекальциферола [127].

Вместе с тем отдельно можно выделить некоторые группы пациентов, требующие дифференцированного подхода (табл. 2) [127].

**Table:** Таблица 2. Алгоритм подбора дозы с лабораторным контролем уровня 25(ОН)D Table 2. Algorithm for dose selection with laboratory monitoring of 25(OH)D levels * — длительный прием препаратов витамина D, особенно высокодозных, у пациентов с деменцией/иными когнитивными нарушениями, должен производиться под контролем родственников/сиделок/иного обслуживающего персонала; ** — рекомендовано определение уровня кальция в крови перед назначением терапевтических доз витамина D и через 2 месяца терапии; *** — препараты витамина D наряду с кальцием входят в любые схемы и режимы лечения остеопороза. Если перед началом патогенетической терапии остеопороза пациент не принимал добавки витамина D и исследование концентрации 25(OH)D в сыворотке крови невозможно, а при этом планируется лечение сильными антирезорбтивными препаратами (золедроновая кислота, деносумаб), рекомендуется назначить умеренную нагрузочную дозу нативного витамина D — 5000 МЕ/сут в течение 10 дней либо однократно 50 000 МЕ, после чего перейти на обычную поддерживающую дозу 800–2000 МЕ/сут. [127–129]. # — рекомендован контроль концентрации витамина D после 2 месяцев приема терапевтических доз витамина D. Контроль следует проводить на 3–4 день после приема последней терапевтической дозы.

Прием витамина D противопоказан	• активный саркоидоз; • другие гранулематозы, сопровождающиеся гиперкальциемией; • гиперпаратиреоз с уровнем общего кальция крови более 2,8 ммоль/л; • пациенты с выявленной генетической мутацией (мутация 24-гидроксилазы)	-
Прием витамина D (определение исходной концентрации витамина D в крови перед назначением лекарственных препаратов витамина D не требуется)	Взрослые 18+	800–4000 МЕ/сут ИЛИ 5000 МЕ через день ИЛИ 50 000 МЕ 1 раз в месяц (сезонность: ноябрь–апрель) Пример: высокодозный витамин D (Девилам) • 5000–15 000–30 000 МЕ в неделю(1–6 таблеток в неделю) или • 50 000 МЕ в месяц(1 таблетка 50 000 МЕ в месяц)
Пожилые 65+	800–4000 МЕ/сут ИЛИ 5000 МЕ через день или 50 000 МЕ 1 раз в месяц(в течение всего года) Например, высокодозный витамин D (Девилам) • 5000–15000–30 000 МЕ в неделю(1–6 таблеток в неделю) или • 50 000 МЕ в месяц(1 таблетка 50 000 МЕ в месяц)
У некоторых пациентов или при наличии определенных заболеваний или состояний для профилактики рекомендуются в 2–3 раза более высокие дозы витамина D по сравнению со здоровыми взрослыми без других факторов риска (но без применения доз витамина D, превышающих 4000–10 000 МЕ/сут): -пациенты с синдромом мальабсорбции; -пациенты с ожирением ИМТ≥30 кг/м²; -пациенты с темной кожей (монголоидной и негроидной расы)	до 4000–5000–10000 МЕ/сут или 50 000 МЕ/раз в 2 недели(курс 8 недель, с последующим контролем уровня витамина D, при достижении целевых значений возможен длительный прием, при условии сохранения ожирения/мальабсорбции) Пример: высокодозный Витамин D (Девилам) • 25–30 000 МЕ в неделю(5–6 таблеток по 5000 МЕ в неделю) или • 50 000 МЕ в 2 недели(1 таблетка 50 000 МЕ в 2 недели)
Пациенты с диагностированным остеопорозом, остеомаляцией и др. костными заболеваниями перед назначением антирезорбтивной терапии	50 000 МЕ однократно, до инициации антирезорбтивной терапии, а далее — в соответствии с выше рекомендованными дозами в комплексе с препаратами для лечения остеопороза Например, Девилам 50 000 МЕ однократно с переводом на поддерживающую терапию
Прием витамина D (требуется определение исходной концентрации витамина D в крови перед назначением лекарственных препаратов витамина D)**#	Пациенты, нуждающиеся в активном скрининге, с высокой вероятностью выявления дефицита витамина D: остеопороз; остеомаляция; лица со скелетно-мышечной болью; хроническая болезнь почек; печеночная недостаточность; синдромы мальабсорбции (например, муковисцидоз, воспалительные заболевания кишечника, бариатрическая хирургия, радиационный энтерит); гиперпаратиреоз; хроническое лечение препаратами, влияющими на метаболизм витамина D (например, противосудорожными, противотуберкулезными препаратами, глюкокортикоидами, лекарствами от СПИДа, противогрибковыми средствами, холестирамином, гиполипидемическими препаратами, орлистатом); хронические аутоиммунные заболевания (например, рассеянный склероз, ревматоидный артрит); беременные и кормящие женщины; пациенты домов престарелых; пожилые люди с падениями или нетравматическими переломами в анамнезе; заболевания, образующие гранулемы, в неактивной форме (например, саркоидоз, туберкулез, гистоплазмоз, бериллиоз, кокцидиомикоз); пациенты с болезнью Альцгеймера и сосудистыми нарушениями в анамнезе; пациенты, придерживающиеся вегетарианства или строгой диеты с целью быстрого снижения веса	При выявленном дефиците витамина D (уровень 25(ОН)D<20 нг/мл): • 50 000 МЕ еженедельно в течение 8 недель внутрь Например, Девилам 50 000 МЕ 1 таблетка в неделю 8 недель с последующим переводом на профилактическую терапию При выявленной недостаточности витамина D (уровень 25(ОН)D≥20 и <30 нг/мл): • 50 000 МЕ еженедельно в течение 4 недель внутрь Например, Девилам 50 000 МЕ 1 таблетка в неделю 4 недели с последующим переводом на профилактическую терапию Следует избегать однократных доз свыше 50 000 МЕ особенно в старшей возрастной когорте пациентов После достижения адекватного уровня витамина D(уровень 25(ОН)D ≥30 нг/мл) у пациентов с ранее выявленным дефицитом/недостаточностью витамина D показан регулярный длительный прием препаратов витамина D в профилактических дозах!

Особое внимание Совет экспертов обратил на внесение в Резолюцию информации, что терапия и профилактика недостаточности/дефицита витамина D должны проводиться лекарственными препаратами, зарегистрированными в ГРЛС, а не биологически активными добавками, поскольку последние не предназначены для лечения и профилактики недостаточности/дефицита витамина D, а являются лишь нутриентами для нормализации пищевого рациона, проходят контроль только при регистрации препарата, но не имеют контроля по мониторингу и регистрации нежелательных явлений (фармаконадзор), и, в соответствии с действующим законодательством, БАД-D в РФ могут применяться в дозах, не превышающих 600 МЕ/сут, что недостаточно для лечения и поддерживающей терапии дефицита и недостаточности витамина D [130].

Эксперты рекомендовали широко использовать препарат Девилам (50 000 МЕ, 5 000 МЕ) в рамках зарегистрированной инструкции по применению — в качестве препарата выбора для профилактики и лечения дефицита/недостаточности витамина D, с учетом быстрого достижения терапевтического эффекта, хорошей переносимости и безопасности препарата, связанной с особым механизмом высвобождения действующего вещества и удобства интермиттирующего приема для достижения большей комплаентности пациентов проводимому лечению.

## ДОПОЛНИТЕЛЬНАЯ ИНФОРМАЦИЯ

Источники финансирования. Работа выполнена по инициативе авторов без привлечения финансирования».

Конфликт интересов. Авторы декларируют отсутствие явных и потенциальных конфликтов интересов, связанных с содержанием настоящей статьи

Участие авторов. Все авторы одобрили финальную версию статьи перед публикацией, выразили согласие нести ответственность за все аспекты работы, подразумевающую надлежащее изучение и решение вопросов, связанных с точностью или добросовестностью любой части работы.
